# A Robust Quadruple Protein-Based Indirect ELISA for Detection of Antibodies to African Swine Fever Virus in Pigs

**DOI:** 10.3390/microorganisms11112758

**Published:** 2023-11-13

**Authors:** Min-Chul Jung, Van Phan Le, Sun-Woo Yoon, Thi Ngoc Le, Thi Bich Ngoc Trinh, Hye Kwon Kim, Jung-Ah Kang, Jong-Woo Lim, Minjoo Yeom, Woonsung Na, Jin-Ju Nah, Ji-Da Choi, Hae-Eun Kang, Daesub Song, Dae Gwin Jeong

**Affiliations:** 1 Bionanotechnology Research Center, Korea Research Institute of Bioscience and Biotechnology, Daejeon 34141, Republic of Korea; minchul@kribb.re.kr (M.-C.J.); ngoc@kribb.re.kr (T.N.L.); kjungah@kribb.re.kr (J.-A.K.); 2 Department of Proteome Structural Biology, KRIBB School of Bioscience, University of Science and Technology, Daejeon 34141, Republic of Korea; 3 Department of Microbiology and Infectious Diseases, Faculty of Veterinary Medicine, Vietnam National University of Agriculture, Hanoi 100000, Vietnam; letranphan@vnua.edu.vn (V.P.L.); trinhthibichngoc.vet@gmail.com (T.B.N.T.); 4 Department of Biological Science and Biotechnology, Andong National University, Andong 36729, Republic of Korea; syoon@andong.ac.kr; 5 Department of Microbiology, College of Natural Sciences, Chungbuk National University, Cheongju 28644, Republic of Korea; khk1329@chungbuk.ac.kr; 6 College of Veterinary Medicine and Research Institute for Veterinary Science, Seoul National University, Seoul 08826, Republic of Korea; nanobiolim@snu.ac.kr (J.-W.L.); virusxnox@snu.ac.kr (M.Y.); 7 College of Veterinary Medicine, Chonnam National University, Gwangju 61186, Republic of Korea; wsungna@jnu.ac.kr; 8 Foreign Animal Disease Division, Animal and Plant Quarantine Agency, Gimcheon 39660, Republic of Korea; nahjj75@korea.kr (J.-J.N.); apjida@korea.kr (J.-D.C.); kanghe@korea.kr (H.-E.K.)

**Keywords:** African swine fever virus, ELISA, quadruple recombinant protein, CD2v, CAP80, p22, p54, porcine, emerging virus, detection

## Abstract

African swine fever (ASF) emerged in domestic pigs and wild boars in China in 2018 and rapidly spread to neighboring Asian countries. Currently, no effective vaccine or diagnostic tests are available to prevent its spread. We developed a robust quadruple recombinant-protein-based indirect enzyme-linked immunosorbent assay (QrP-iELISA) using four antigenic proteins (CD2v, CAP80, p54, and p22) to detect ASF virus (ASFV) antibodies and compared it with a commercial kit (IDvet) using ASFV-positive and -negative serum samples. The maximum positive/negative value was 24.033 at a single antigen concentration of 0.25 μg/mL and quadruple ASFV antigen combination of 1 μg/mL at a 1:100 serum dilution. Among 70 ASFV-positive samples, 65, 67, 65, 70, 70, and 14 were positive above the cut-offs of 0.121, 0.121, 0.183, 0.065, 0.201, and 0.122, for CD2v, CAP80, p54, p22-iELISA, QrP-iELISA, and IDvet, respectively, with sensitivities of 92.9%, 95.7%, 92.9%, 100%, 100%, and 20%, respectively, all with 100% specificity. The antibody responses in QrP-iELISA and IDvet were similar in pigs infected with ASFV I. QrP-iELISA was more sensitive than IDvet for early antibody detection in pigs infected with ASFV II. These data provide a foundation for developing advanced ASF antibody detection kits critical for ASF surveillance and control.

## 1. Introduction

African swine fever (ASF) is a severe highly infectious disease that affects domestic pigs and wild boars in many countries. The ASF virus (ASFV) has a negative economic impact on the pig-farming industry due to the need for animal slaughter, high mortality rates, quarantine restrictions, and pork import and export regulations [1]. ASFV is a large icosahedral cytoplasmic virus with a diameter of approximately 200 nm and is the only virus in the *Asfaviridase* family [2,3,4,5]. The genome size of ASFV is approximately 175,000–195,000 bp, arranged as large double-stranded DNA with 150 to 200+ open reading frames [5,6]. The repeated resurgence of ASF has caused an economic crisis in the pig-farming industry in several African and European countries [7,8].

The ASFV epidemic in Asia and Southeast Asia has been a major challenge for the region since ASFV was first reported in China in 2018 [9]. ASFV has spread to over 10 countries: Vietnam, Cambodia, Laos, Myanmar, Thailand, the Philippines, Indonesia, Malaysia, Mongolia, and Korea. The virus is thought to have spread to Asia from Europe in 2007, and it has since become endemic in many regions. As the pork industry is a major economic sector in many Asian countries, the ASFV can cause significant losses to the industry by killing pigs and reducing productivity. ASFV can also lead to the closure of pork processing plants and other businesses that rely on pork products. Thus, the ASFV can disrupt food supply chains by reducing the supply of pork products, leading to shortages and higher prices of pork products [10]. In China, the number of cases has declined in recent years, and ASFV infection is currently largely under control. However, ASFV remains a threat, and new outbreaks are still reported [10]. In Vietnam, the number of ASF outbreaks has increased recently, and the infection has spread to most of the country [11]. The number of outbreaks is increasing in Cambodia, Laos, Myanmar, Thailand, the Philippines, Indonesia, and Malaysia, and the governments of these countries are taking the lead in infection control [12]. Because there is no cure or treatment for ASFV, developing a more effective vaccine is critical, but current commercial vaccines have extremely limited effects [13]. Furthermore, early serological diagnosis of ASFV infection should be prioritized to prevent virus spread to other herds, because clinical signs are not observed in the early stages of infection or are indistinguishable from those of other viral infections [14].

The World Organization for Animal Health (WOAH) and many research groups recommend using enzyme-linked immunosorbent assay (ELISA) for serological diagnosis of ASFV infection. Several commercial ASFV indirect ELISA (iELISA) kits are available worldwide, including Ingenasa (INGEZIM ASF-R, Ingenasa, Madrid, Spain), Ringbio (ASFV Ab ELISA Kit, Ringbio, Beijing, China), Svanovir (SVANOVIR ASFV-Ab, INDICAL, Uppsala, Sweden), and IDvet (ID Screen African Swine Fever Indirect ELISA, IDvet, Grabels, France). These serological diagnostic kits are suitable for large-scale testing, and they have different sensitivities for anti-ASFV antibodies depending on their species and the number of detection antigens [15]. Various approaches are currently being studied to increase the sensitivity using different antigens, including CD2v [16], CAP80 [17], p72 [18], p22 [19,20], p30 [18,19,20,21,22], p54 [22,23], pp62 [24,25], p15 [26], p11.5 [27], and I329L [28]. In particular, CD2v(pEP402R) is a transmembrane protein in the outer envelope that is expressed in the late stage of infection. The glycosylated extracellular N-terminal domain, which is highly homologous to the CD2v protein of the host cell, plays an essential role in viral spread through the adsorption of erythrocytes in infected cells [29,30]. CAP80(pB602L) is a nonstructural protein that is also expressed in the late stage of infection and plays an important role as a chaperone protein in the structural formation of other capsid proteins [31]. P54(pE183L) is a strongly antigenic outer envelope protein of ASFV, and p22(pK177R) is an early-stage structural protein in the inner envelope. These two proteins are involved in viral entry, thus playing an important role in viral infection [32,33,34,35,36].

In this study, we developed and evaluated a quadruple recombinant-protein iELISA (QrP-iELISA) using a combination of four proteins of ASFV genotype II (ASFV II), the N-terminal domain of CD2v, full-length CAP80, and transmembrane domain truncated p54 and p22, with naturally and experimentally infected pig serum samples. Moreover, we compared our QrP-iELISA kit to a commercial ELISA kit. Our results provide essential insights into the spread of ASFV, which can be prevented and controlled early by selecting a more sensitive and specific antigen combination target that binds to antibodies to diagnose ASFV infection.

## 2. Materials and Methods

### 2.1. Serum Sample Collection and Virus Samples

ASFV-positive and -negative serum samples were collected from 80 breeding sows and fattening pigs who displayed signs of infection (high fever, anorexia, vomiting, lethargy, respiratory distress, coughing, and disseminated cyanosis) from various locations in Northern Vietnam (Hanoi, Phu Tho, Vinh Phuc, and Hung Yen Province), from September to November 2019 [37]. ASFV infection was confirmed using a commercial real-time PCR kit, the VDx^®^ ASFV qPCR kit (Median Diagnostics Inc., Chuncheon, Gangwon-do, Republic of Korea). This test amplifies a target sequence in the ASF viral B646L (p72) genome. Of the 80 serum samples from pigs with suspected ASFV infection, 70 were determined as positive for ASFV II with cycle threshold values between 14 and 35. ASFV was not detected in the 10 remaining serum samples (Table S1). Additionally, 16 ASFV-negative serum samples were collected from domestic pigs in South Korea before the ASFV outbreak. All positive and negative serum samples in RT-PCR testing were used as positive and negative reference tests, respectively.

Two ASFV strains were used for the experimental infection study. OURT 88/3, a low-virulence ASFV genotype I (ASFV I) strain provided by the European Union and FAO reference laboratory for ASF (INIA-CISA, Madrid, Spain), and Korea/Pig/Paju1/2019, a highly virulent ASFV II strain from the first ASF outbreak in domestic pigs in South Korea, were propagated in porcine alveolar macrophages [19,38,39].

### 2.2. Cloning, Expression, and Purification of Recombinant CD2v, CAP80, p54, and p22 Proteins

The soluble CD2v, CAP80, p54, and p22 proteins involved in viral infection, showing high antigenicity and mainly located in the outer and inner envelope of ASFV, were selected through expression screening analysis of ASFV structural proteins. A combination of these four proteins was used for iELISA against ASFV originating from the China/2018/AnhuiXCGQ genome sequence (GenBank: MK128995.1). The genes for protein expression were then amplified using PCR with the primers listed in Table S2. The PCR products of CD2v-N spanning amino acid positions 20–197 and p22 spanning amino acid positions 32–177 were cloned into the baculovirus transfer vector pAcGP67A (BD Biosciences, San Diego, CA, USA). A signal peptide for protein secretion was added at the N-terminal end, and a 6-histidine (6xHis) tag was added at the C-terminal end. The CAP80 spanning amino acid positions 1–530 was cloned into the bacterial expression vector pET-28a (Novagen, San Diego, CA, USA), and p54 spanning amino acid positions 59–184 was also cloned to pET-28a fused to a 6xHis-tagged maltose-binding protein (MBP) at the N-terminal end. A tobacco etch virus protease (TEVp) site was inserted between MBP and p54 to cleave and remove MBP.

The pAcGP67A-CD2v-N and pAcGP67A-p22 vectors were transfected into *Spodoptera frugiperda* (Sf9) insect cells (Thermo Fisher Scientific, Waltham, MA, USA) with linearized baculovirus DNA (AB Vector, San Diego, CA, USA) to express the CD2v-N and p22 proteins, according to the manufacturer’s instructions, to generate recombinant baculovirus. Initial recombinant virus stocks were harvested 60–72 h after transfection, and each subsequent recombinant baculovirus passage was obtained from the recombinant virus stocks at 28 °C for 3 days. The fifth passage, as a high titer of recombinant baculovirus, was used to express CD2v-N and p22 proteins in High Five (BTI-Tn-5B1-4, Hi5) cells (Thermo Fisher Scientific, Waltham, MA, USA), and secreted proteins were analyzed via Western blotting of the clarified culture medium, and purified by Ni-NTA Superflow(Qiagen) and Superdex 200 pg 16/600 column (Cytiva, Marlborough, MA, USA).

The pET-28a-CAP80 and pET-28a-MBP-p54 were transformed into *Escherichia coli* BL21(DE3)-RIL cells and cultured at 37 °C, and protein expression was induced by adding 0.2 mM isopropyl β-D-1-thiogalactopyranoside at 18 °C for 18 h. The *E. coli* cell lysates that expressed CAP80 protein were loaded into a Ni-NTA Superflow column and eluted using imidazole buffer. The collected CAP80 protein was then purified using a Q-sepharose ion exchange column (Cytiva) and a Superdex 200 pg 16/600 column (Cytiva). The *E. coli* cell lysates that expressed 6xHis-tagged MBP-p54 fusion protein were loaded into a Ni-NTA Superflow column and eluted. Next, the fusion protein was incubated with TEVp to remove the 6xHis-tagged MBP and then loaded into a Ni-NTA Superflow column to collect the unbound p54 protein. Finally, the unbound p54 protein was purified using Superdex 75 16/600 chromatography (Cytiva). The purity and concentration of purified proteins were subsequently confirmed via SDS-PAGE and Bradford assay (Bio-Rad Laboratories, Hercules, CA, USA).

### 2.3. Detection of Antibody Using Single Recombinant Protein (SrP)- and QrP-iELISA

Aliquots of 100 μL of SrP (25 ng/100 μL per well) and QrP (25 ng of each protein, and 100 ng/100 μL of the four recombinant proteins combined) in 0.2 M sodium carbonate buffer (pH 9.6) were incubated in 96-well microplates at 4 °C overnight. The plates were washed three times with PBS (pH 7.5) containing 0.05% (*v*/*v*) Tween 20 (PBST). Next, the plates were blocked with PBST containing 5% (*w*/*v*) skim milk (PBST-M) as a blocking buffer at 37 °C for 1 h, and then the blocking buffer was removed. A 100 μL aliquot of serum sample diluted at a ratio of 1:100 in PBST-M was added to the plate and incubated at 37 °C for 1 h, followed by washing three times with PBST. Horseradish peroxidase (HRP)-conjugated anti-pig IgG (Abcam, Cambridge, MA, USA) was diluted to 1:20,000 in PBST-M and added to each well. After incubation at 37 °C for 30 min, the plates were washed three times with PBST. Finally, the reaction was developed by adding 100 μL of 3,3′,5,5′-tetramethylbenzidine (TMB) as substrate per well at 37 °C for 3 min. The reaction was then terminated by adding 50 μL of 1 N sulfuric acid per well, and the optical density at 450 nm (OD_450_) was measured using a plate reader (Victor III; PerkinElmer, Waltham, MA, USA).

### 2.4. Serum Dilution Conditions and Cut-Off Value for iELISA

The optimal iELISA conditions were determined by coating the wells with QrP at a concentration of 1 μL/mL and diluting ASFV-positive and -negative serum samples at 1:10, 1:100, and 1:1000. The criteria for determining the optimal condition were that the OD_450_ of the positive serum sample exceeded 1, and the OD_450_ ratio (P/N value) of the positive and negative serum samples had the highest values of the dilutions tested. Twenty-six ASFV-negative serum samples were measured using individual SrP-iELISA, QrP-iELISA, and IDvet kits to calculate the cut-off value. The OD_450_ values were measured and statistically analyzed to calculate the mean and standard deviation (SD), and the cut-off values were set at the means + 3SD. OD_450_ values above the cut-off value were considered positive, and those below the cut-off value were considered negative.

### 2.5. Sensitivity and Specificity in Naturally and Experimentally Infected Serum Samples

The sensitivity and specificity of individual SrP-iELISA, QrP-iELISA, and IDvet ELISA kits (ID Screen African Swine Fever Indirect ELISA, IDvet, Grabels, France) were determined by comparing a total of 70 ASFV-positive and 26 negative serum samples to the cut-off values. Depending on the real-time PCR results for ASFV as a reference test, sensitivity was determined by the ability to detect the positivity of the screening test based on the true positive rate. Specificity was determined by the ability to detect the negativity of the screening test based on the true negative rate. The following formulas were used to calculate the sensitivity and specificity:

Sensitivity = 100 × (number of positive test samples)/(total number of positive samples using the reference test)

Specificity = 100 × (number of negative test samples)/(total number of negative samples using the reference test)

Differences in antibody responses to CD2v, CAP80, p54, and p22 proteins were analyzed using serum samples collected from experimentally ASFV-infected pigs. Experimental infection was performed in an Animal Biosafety Level 3 facility at the Animal and Plant Quarantine Agency (APQA) in Gimcheon, South Korea. First, five 8-week-old pigs in Group 1 (8-6 to 8-10) were infected with 10^4^ × tissue culture infective dose 50%/mL of ASFV OURT 88/3 strain (ASFV I), and five additional pigs of the same age in Group 2 (7-1 to 7-5) were infected with 10 × 50% hemadsorption dose of Korea/Pig/Paju1/2019 strain (ASFV II). In each infected group, serum samples were collected on 0, 3, 5, 7, 8, 9, 11, 12, 13, 14, and 28 days post-infection (DPI). All collected serum samples were analyzed using individual SrP-iELISA, QrP-iELISA, and the commercial ID vet ELISA kit.

### 2.6. Statistical Analysis

Statistical analyses were performed using GraphPad Prism 8.0 (GraphPad Software, San Diego, CA, USA). In the analysis of the ELISA results, groups were compared using *t*-tests, and two-tailed *p* values < 0.05 were considered statistically significant.

## 3. Results

### 3.1. Recombinant Protein Production

**Figure 1:**
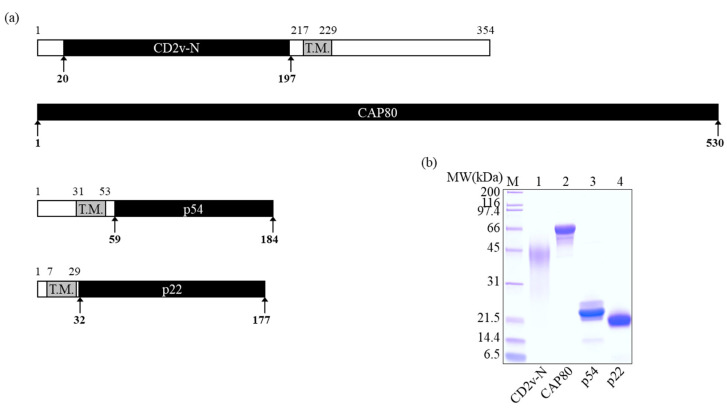
Construction and purification of quadruple recombinant proteins (QrPs). (**a**) Primary protein structure of QrPs. (**b**) SDS-PAGE analysis of purified recombinant proteins. Lane M: protein molecular size marker; lane 1: CD2v-N; lane 2: CAP80; lane 3: p54; lane 4: p22. The gray bars labeled “T.M.” represent the transmembrane region. Expressed and purified QrP domains are indicated by the numbers with arrows and are represented as black bars.

The full-length gene-coding sequences of CD2v(pEP402R), CAP80(pB602L), p54(pE183L), and p22(pK177R) were obtained from the genome sequence of China/2018/AnhuiXCGQ (GenBank: MK128995.1), which was reported in China in September 2018 [40] and had 100% amino acid sequence identity to CD2v, CAP80, p54, and p22 with Eastern European ASFV II strains, respectively. According to the secondary structure of each protein, the optimal protein domain for protein expression was designed considering the hydrophilicity, flexible loops, and transmembrane regions. Each recombinant protein was designed to increase production efficiency by truncating the transmembrane region based on the bioinformatics analysis (Figure 1a). CD2v-N and p22 were expressed in a baculovirus expression system, and CAP80 and p54 were expressed in a prokaryotic cell expression system, considering expression location and protein modification within the ASFV. All proteins were expressed as soluble and purified to high purity, which was confirmed using SDS-PAGE (Figure 1b). The calculated molecular weights of the purified CN2v-N, CAP80, p54, and p22 were 21,934, 65,110, 14,827, and 18,070 Da, respectively.

### 3.2. Optimal Serum Dilution Condition for QrP-ELISA

The optimal serum dilution condition of QrP-ELISA was determined from the OD_450_ value of the positive and negative serum samples with a maximum value (P/N ratio of 24.033) at a dilution of QrP antigen and serum of 1 μL/mL and 1:100, respectively (Table 1). Thus, for QrP-iELISA, a total of 100 ng of QrP antigen and 25 ng of each SrP (CD2v-N, CAP80, p54, and p22) antigen were used to coat each well in a ratio of 1:1:1:1, and the optimal serum dilution was performed at 1:100. The conditions of blocking buffer, ASFV antiserum incubation time and temperature, dilution ratio of secondary HRP-conjugated antibody, and TMB reaction time were also optimized to provide the highest P/N ratio.

### 3.3. Setting the Cut-Off Value

The mean and SD of each iELISA panel were obtained with the ASFV-negative serum samples (n = 26) (Table S3). The cut-off value of each ELISA was calculated as the mean + 3SD and set to 0.201 of QrP-iELISA, 0.121 of CD2v-N-ELISA, 0.121 of CAP80-iELISA, 0.183 of p54-iELISA, 0.065 of p22-iELISA, and 0.122 of IDvet.

### 3.4. Comparison of SrP-and QrP-iELISA with Commercial ELISA in Serum Samples from Pigs with Natural Infection

**Figure 2:**
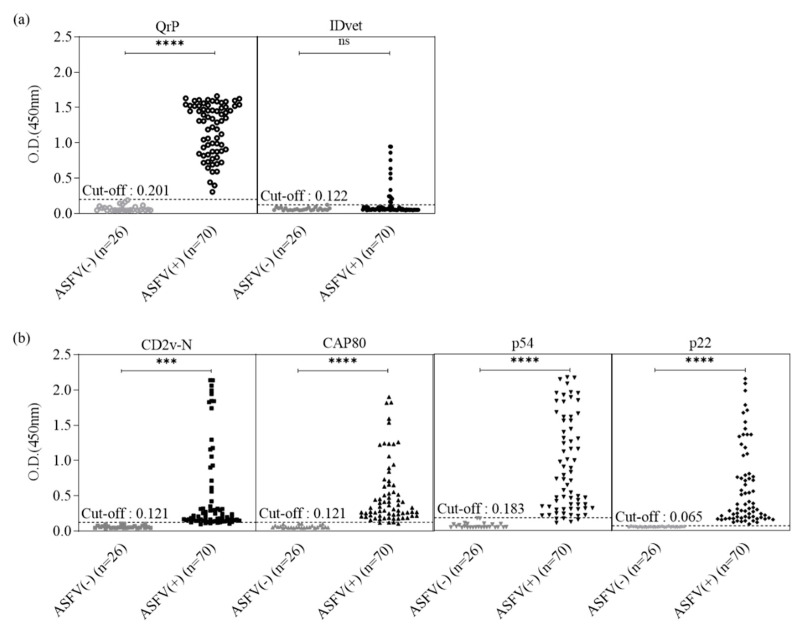
Evaluation of SrP- and QrP-iELISAs and the commercial kit. (**a**) Comparison between QrP-iELISA and the commercial IDvet kit. (**b**) Comparison between SrP-iELISAs. iELISA, indirect enzyme-linked immunosorbent assay; SrP, single recombinant protein; ns: not significant; *** *p* < 0.001; **** *p* < 0.0001.

We used 70 ASFV-positive and 26 ASFV-negative serum samples to evaluate the sensitivity and specificity of SrP-iELISA, QrP-iELISA, and an IDvet ELISA kit (Figure 2). Of the 70 ASFV-positive samples, QrP-iELISA showed that all 70 serum samples were positive, with a cut-off value of ≥0.201, indicating 100% sensitivity, whereas only 14 of the 70 positive samples tested positive using IDvet ELISA, with a cut-off value of ≥0.122. IDvet ELISA showed a sensitivity of 20%, which was one-fifth of that of the QrP-ELISA (Figure 2a, Table 2). For CD2V-N, CAP80, p54, and p22 SrP-iELISA, among 70 ASFV-positive serum samples, 65, 67, 65, and 70 samples were positive using cut-off values of ≥0.121, ≥0.121, ≥0.183, and ≥0.065, for a sensitivity of 92.9%, 95.7%, 92.9%, and 100%, respectively (Figure 2b, Table 2). Each SrP- and QrP-iELISA, as well as the IDvet kit, showed 100% specificity, because all negative samples detected by the reference test were accurately determined as negative (Table 2).

### 3.5. Comparing SrP-ELISA, QrP-iELISA, and Commercial ELISA Testing of Serum Samples from Experimentally Infected Pigs

**Figure 3:**
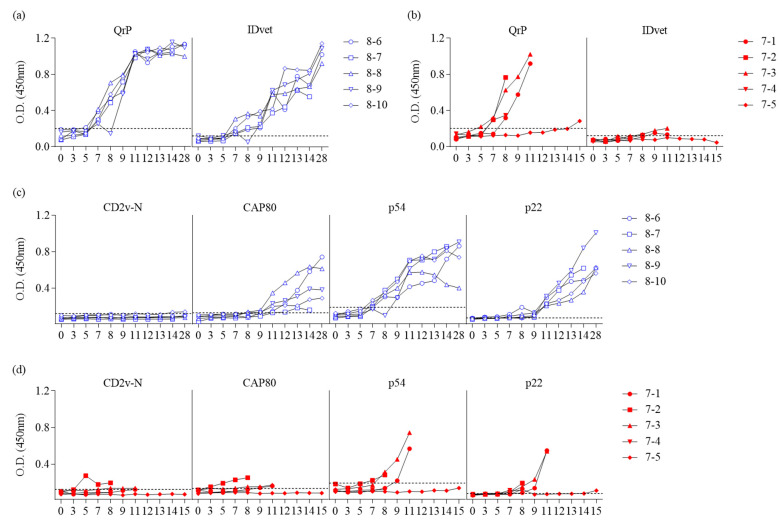
Comparison of antibody responses to SrP and QrP in experimentally infected pig serum samples. (**a**) Comparison of QrP-ELISA and IDvet results using serum samples of pigs infected with ASFV I. (**b**) Comparison of QrP-ELISA and IDvet results using serum samples of pigs infected with ASFV II. (**c**) Comparison of SrP-ELISA results using serum samples of pigs infected with ASFV I. (**d**) Comparison of SrP-ELISA results using serum samples of pigs infected with ASFV II. The *X*-axis of each plot shows the days post-infection (DPI), and the *Y*-axis shows the OD_450_ value. The horizontal dashed lines show the OD_450_ threshold for positivity.

The differences in antibody response to SrPs and QrP were evaluated using serum samples collected from 0 to 28 DPI from 10 pigs experimentally infected with ASFV. Five pigs were infected with ASFV I (OURT 88/3), and five were infected with ASFV II (Korea/Pig/Paju1/2019) (Figure 3).

Serum samples were collected from the 10 experimentally infected pigs to create antisera. Antibody detection was stratified by DPI. Antibody responses to each CD2v-N, CAP80, p54, p22, and QrP-iELISA were compared with those measured using the IDvet kit (Figure 3). In Group 1, the first antibody response was detected in one pig (8-6) at 5 DPI using QrP-iELISA, and all five pigs showed a positive antibody response at 7 DPI. The IDvet kit also showed a positive response at 5 DPI. Starting with one pig (8-9), all five tested positive for antibodies at 7 DPI using the IDvet kit (Figure 3a).

In Group 2, four of the five pigs (7-1 to 7-4) died at 8 to 11 DPI, and one pig (7-5) survived. The first antibody response was detected in one pig (7-3) at 3 DPI using QrP-iELISA, followed by three pigs at 7 DPI, and four pigs at 8 DPI. Two pigs subsequently died. The only surviving pig (7-5) showed a positive antibody response at 15 DPI. Using the IDvet kit, two pigs tested positive for antibodies at 8 DPI, two pigs died after 8 DPI without testing positive, and the last remaining pig showed a negative antibody response until 14 DPI. In Group 2, the OD_450_ value of the antigen against the antiserum of the pigs that tested positive for antibodies was close to 1 using QrP, which was much higher than the OD_450_ value obtained using the IDvet kit (Figure 3b).

In Group 1, although all four SrP (CD2v-N, CAP80, p54, and p22)-iELISAs could detect an antibody response in the antiserum, only one pig tested positive on CD2v-N-iELISA at 14 DPI. Using the CAP80-iELISA, two pigs were antibody-positive at 8 DPI, and five pigs were antibody-positive at 11 DPI. Using the p54-iELISA, two pigs tested positive at 7 DPI and 8 DPI, and five pigs at 9 DPI were all positive for antibodies. Among the four SrP-iELISAs, positive antibodies were detected by the p22-iELISA earliest at 3 DPI, and positive antibodies were detected in all pigs at 9 DPI. Among the five experimentally infected pigs in Group 1, only one pig (8-7) died at 28 DPI (Figure 3c).

In Group 2, antibody positivity was detected in one pig (7-2) at 5 DPI and one pig (7-3) at 7 DPI using CD2v-N-iELISA. Two pigs (7-2, 7-4) died at 9 DPI, only one of which tested positive for antibodies using CD2v-N-iELISA. Two pigs were detected as antibody-positive at 3 DPI using the CAP80-iELISA, and one pig tested as antibody-positive at 7 DPI and one tested positive at 8 DPI using the p54-iELISA. Using the p22-iELISA, one pig tested antibody-positive at 3 DPI, two pigs tested antibody-positive at 7 DPI, and all five pigs tested antibody-positive at 8 DPI. Of the five pigs in Group 2, four had died by 12 DPI, and the remaining pig (7-5) tested positive at 14 and 15 DPI using p22-iELISA (Figure 3d).

The number of seropositive pigs and the antibody detection rate (%) among all infected pigs by date after infection in Groups 1 and 2 using QrP-iELISA, the IDvet kit, and SrP-iELISAs are shown in Table 3.

## 4. Discussion

In this study, we developed QrP-iELISA using four ASFV proteins involved in virus entry and infection for serological diagnosis of ASFV infection. A QrP and four SrPs were successfully designed, expressed, and purified using the *E. coli* and baculovirus expression system to maintain structural stability and solubility. CD2v is an important surface structural protein of ASFV that binds specifically to CD2 receptors on the surface of porcine red blood cells [16,41]. Infection with wild-type ASFV produces specific antibodies that recognize the CD2v protein. CD2v was purified by secreting the N-terminal extracellular domain ranging from 20 to 197, excluding the transmembrane and intracellular domains, to maintain post-translational modification and glycosylation using the baculovirus expression system. Highly glycosylated CD2v-N, which has a structure similar to that of the native viral protein, appeared as a smeared band at about twice the original molecular weight in SDS-PAGE analysis after purification [16,41]. The p22 protein ranging from 32 to 177 was also secreted using the baculovirus expression system by removing the transmembrane domain at the N terminus, which yielded a considerably greater amount and purity of p22 owing to disulfide-bond formation for a more stable and rigid protein structure than that expressed in *E. coli*. The full-length CAP80 protein ranging from 1 to 530 was expressed and purified well in *E. coli*, but the p54 protein ranging from 59 to 184 was slightly soluble by itself in *E. coli* and was improved to express as a fusion protein with the MBP protein, resulting in soluble protein after MBP was removed with TEVp. Thus, the design of the recombinant protein and expression system for antigen is critical; if the antigen used in ELISA, which detects antibodies produced after ASFV infection, is closer to the three-dimensional protein structure of the natural viral protein, antibody detection would improve.

In most previous studies for the detection of antibodies produced after ASFV infection, iELISA was developed and evaluated mainly using single, double, or triple ASFV antigens. Many ELISAs are being developed in laboratories worldwide, with single antigen coating concentrations ranging from 0.4 to 1.2 μg/mL, serum dilutions ranging from 1:10 to 1:600, and P/N ratios for antiserum ranging from 8.3 to 55.73 [17,18,19,20,22,42]. Notably, we developed and optimized QrP-iELISA using a combination of four ASFV antigens. Our QrP-iELISA optimized the concentration of the coating antigen and the dilution condition of the antisera; the maximum P/N value was 24.033 at a concentration of the single antigen coating of 0.25 μg/mL, and a concentration of the quadruple ASFV antigen combination of 1 μg/mL, with a serum dilution of 1:100. The QrP-iELISA showed high sensitivity and serum dilution ratio compared with that of the commercial IDvet ELISA kit and previously studied antigens. Because the types of each antigen and ELISA conditions are different, direct comparison of the optimal conditions is complicated, but the performance can be compared under the same ASFV serum sample conditions. We suggest that the combination and ratio of antigens used in ELISA, as well as the antigens selected and their high-quality preparation, are important factors for performance.

In the present study, we collected 70 ASFV-positive and 26 ASFV-negative naturally infected serum samples from Vietnam and Korea and validated SrP-ELISA and QrP-ELISA by comparing them with the IDvet ELISA kit. QrP-iELISA detected antibodies in all 70 positive serum samples with high OD_450_ values, but IDvet detected antibodies only in 14 of the 70 positive serum samples. Our results revealed that QrP-iELISA could detect positives more effectively with sensitivity five times higher than that of the IDvet ELISA under the same conditions. Each SrP-iELISA also had a high sensitivity of 92.9–100%, and QrP-iELISA had a higher OD_450_ value (>0.5) than those of the SrP-iELISAs using ASFV-positive serum samples, indicating that the combination of four proteins was more effective in detecting antibodies than single proteins as the coating antigen. All SrP-iELISAs, the QrP-iELISA, and the IDvet ELISA showed 100% specificity for negative serum samples without cross-reactivity. Moreover, our four antigens expressed at different stages after ASFV infection enabled antibody detection during the entire post-infection period [29,30,31,32,33,34,35,36].

The IDvet kit used triple proteins, p72, p62, and p30, as a coating antigen, which originated from ASFV I and had 99.54%, 99.81%, and 97.42% identity, respectively, with the amino acid sequences of ASFV II proteins. In contrast, QrP-iELISA used the QrPs CD2V-N, CAP80, p54, and p22 as coating antigens, which originated from ASFV II and had 52.25%, 98.11%, 96.03%, and 98.63% identity, respectively, with amino acid sequences of the ASFV I protein. CD2v-N-iELISA had the most delayed and lowest ability to detect an antibody response in pigs infected with ASFV I OURT 88/3 due to the low amino acid sequence identity of 52.25% with ASFV I CD2v-N. Our results revealed that QrP-iELISA and the IDvet kit had similar antibody responses in pigs infected with ASFV I. In pigs infected with ASFV II, QrP-iELISA, which comprises ASFV II antigens, performed better than the IDvet kit in detecting antibodies and could detect antibodies earlier. Overall, QrP-iELISA performed better than the IDvet kit in detecting antibodies in pigs infected with either ASFV I or II.

In conclusion, developing a sensitive and effective serological diagnostic ELISA for early and rapid detection of ASFV infection is important for controlling its spread. We confirmed that, compared with the commercial IDvet kit, our SrP-ELISA and QrP-iELISA kits were more sensitive and could detect ASFV I and II antibodies in serum samples earlier in the course of infection. These data are essential for developing advanced ASF antibody detection ELISA kits, which are critical for the large-scale surveillance needed to monitor and control the spread of ASF outbreaks.

## Figures and Tables

**Table 1:** Determination of the optimal serum dilution according to the P/N ratio.

Serum Dilution	OD450 nm		P/N Ratio
1:10	P	1.073	12.056
	N	0.089	
1:100	P	1.022	24.033
	N	0.042	
1:1000	P	0.585	15.000
	N	0.039	

P: Positive control serum, N: negative control serum.

**Table 2:** Sensitivity and specificity of the SrP-iELISA, QrP-iELISA, and commercial ELISA (IDvet) used to detect antibodies against African swine fever virus (ASFV) in this study.

Test	Positive (n = 70)	Sensitivity (%)	Negative (n = 26)	Specificity (%)
QrP-iELISA	70	100.0	26	100.0
IDvet kit	14	20.0	26	100.0
CD2v-N-ELISA	65	92.9	26	100.0
CAP80-ELISA	67	95.7	26	100.0
p54-ELISA	65	92.9	26	100.0
p22-ELISA	70	100.0	26	100.0

**Table 3:** Differences in the antibody response in SrP-ELISAs, QrP-ELISA, and commercial kit according to DPI.

DPI	QrP		IDvet		CD2v-N		CAP80		p54		p22	
	Positive/ Total	%	Positive/ Total	%	Positive/ Total	%	Positive/ Total	%	Positive/ Total	%	Positive/ Total	%
	Group 1: Infected with ASFV OURT 88/3 (Genotype I)											
0	0/5	0	0/5	0	0/5	0	0/5	0	0/5	0	0/5	0
3	0/5	0	0/5	0	0/5	0	0/5	0	0/5	0	2/5	40
5	1/5	20	1/5	20	0/5	0	0/5	0	0/5	0	1/5	20
7	5/5	100	5/5	100	0/5	0	0/5	0	2/5	40	4/5	80
8	4/5	80	4/5	80	0/5	0	2/5	40	4/5	80	4/5	80
9	5/5	100	5/5	100	0/5	0	2/5	40	5/5	100	5/5	100
11	5/5	100	5/5	100	0/5	0	5/5	100	5/5	100	5/5	100
12	5/5	100	5/5	100	0/5	0	5/5	100	5/5	100	5/5	100
13	5/5	100	5/5	100	0/5	0	5/5	100	5/5	100	5/5	100
14	5/5	100	5/5	100	1/5	20	5/5	100	5/5	100	5/5	100
28	4/4	100	4/4	100	1/4	25	4/4	100	4/4	100	4/4	100
	Group 2: Infected with ASFV Korea/Pig/Paju1/2019 (Genotype II)											
0	0/5	0	0/5	0	0/5	0	0/5	0	0/5	0	0/5	0
3	0/5	0	0/5	0	0/5	0	2/5	40	0/5	0	1/5	20
5	1/5	20	0/5	0	1/5	20	1/5	20	0/5	0	1/5	20
7	3/5	60	0/5	0	2/5	40	2/5	40	1/5	20	3/5	60
8	4/5	80	2/5	40	2/5	40	2/5	40	2/5	40	5/5	100
9	2/3	67	2/3	67	1/3	33	2/3	67	2/3	67	2/3	67
11	2/3	67	2/3	67	1/3	33	2/3	67	2/3	67	2/3	67
12	0/1	0	0/1	0	0/1	0	0/1	0	0/1	0	0/1	0
13	0/1	0	0/1	0	0/1	0	0/1	0	0/1	0	0/1	0
14	0/1	0	0/1	0	0/1	0	0/1	0	0/1	0	1/1	100
15	1/1	100	0/1	0	0/1	0	0/1	0	0/1	0	1/1	100

## Data Availability

All data produced and analyzed in this study are included in the article.

## Supplementary Materials

The following supporting information can be downloaded at: https://www.mdpi.com/article/10.3390/microorganisms11112758/s1, Table S1: Validation of naturally ASFV-infected serum samples; Table S2: List of primer sequences in this study for PCR amplification; Table S3: The mean, standard deviation, and cut-off value of negative sera.
