# Supplementary material for: A Robust Quadruple Protein-Based Indirect ELISA for Detection of Antibodies to African Swine Fever Virus in Pigs
Source: Microorganisms. 2023 Nov 13;11(11):2758. doi: 10.3390/microorganisms11112758 (PMC10672928; doi:10.3390/microorganisms11112758)
Supplement: Supplementary file 1 [file microorganisms-11-02758-s001.zip › microorganisms-2691064-supplementary.pdf]

Table S1. Validation of naturally ASFV-infected serum samples

No.	Ct value	Collected date	Location	No.	Ct value	Collected date	Location
1	N.D [a]	2019-09-19	Phu Tho	41	16.07	2019-10-25	Hung Yen
2	N.D			42	16.89		
3	N.D			43	18.97		
4	N.D	2019-11-01		44	27.35		
5	N.D			45	14.73		
6	N.D			46	26.27		
7	N.D	2019-10-25	Hung Yen	47	15		
8	N.D			48	15.93		
9	N.D			49	24.04		
10	N.D	2019-11-01		50	14.66		
11	14.29	2019-09-19	Phu Tho	51	15.58		
12	26.4			52	19.1		
13	22.05			53	26.53		
14	23.27			54	16.51		
15	16.53			55	14.99		
16	28.32			56	24.36		
17	18.19			57	13.96		
18	28.93			58	14.42		
19	17.61			59	18.08		
20	18.44	2019-09-23	Vinh Phuc	60	14		
21	15.62			61	30.54		
22	19.54			62	12.13		
23	16.71	2019-10-23	Hung Yen	63	17.06		
24	15.68			64	17.46		
25	24.7			65	14.04		
26	23.39			66	29.75		
27	15.82			67	28.78		
28	16.36			68	14.45	2019-11-01	
29	26.78	2019-10-24	Ha Noi	69	19.44		
30	14.16			70	14.91		
31	35.07			71	15.94		
32	28.35			72	13.67		
33	15.27			73	15.2		
34	15.2	2019-10-25	Hung Yen	74	15.27		
35	14.43			75	14.37		
36	27.39			76	14.14		
37	14.24			77	15.95		
38	14.94			78	16.24		
39	14.07			79	15.06		
40	28.34			80	15.52		

80 field serum samples from Vietnam were identified as ASFV negative (n = 10) and positive (n = 70) by real-time PCR kit, the VDx® ASFV qPCR kit (VDx® ASFV qPCR, Median Diagnostics, Korea)

[a] N.D : Non detected

Table S2. List of primer sequences in this study for PCR amplification

Primer	Sequence
CD2v-N forward	CCGGAATTCGGTTAGTTTTAATAAAACAATAATTTTAGATAG
CD2v-N reverse	GGAAAAGCGGCCGCGTTAGATGACAATGTTAAATAAGTACAAT
CAP80 forward	CCGGAATTCATGGCAGAATTTAATATTGATGAGC
CAP80 reverse	GGAAAAGCGGCCGCTTACAATTCTGCTTTTG
p54 forward	CGCGGATCCGCTGCTGCTATTGAGGAGGA
p54 reverse	CCGCTCGAGTTACAAGGAGTTTTCTAGGTCTTTATGC
p22 forward	CGCGGATCCCCAACAACCACCGAAAAAGGTC
p22 reverse	GGAAAAGCGGCCGCTGCATGTTTATGATTTCTAGGTAAG

[a] Underlined bases indicate restriction enzyme recognition sites.

Table S3. The mean, standard deviation, and cut-off value of negative sera

	Mean ($\overline{X}$)	Standard deviation (SD)	Cut-off value ($\overline{X}$ + 3SD)
QrP-iELISA	0.074	0.042	0.201
IDvet	0.061	0.020	0.122
CD2v-N-iELISA	0.060	0.020	0.121
CAP80-iELISA	0.061	0.020	0.121
p54-iELISA	0.071	0.037	0.183
p22-iELISA	0.051	0.005	0.065

ELISA, enzyme-linked immunosorbent assay; iELISA, indirect enzyme-linked immunosorbent assay; QrP quadruple recombinant protein; SrP, single recombinant protein.
